# The NEO-1 gene may serve as a potential biomarker for predicting acute myocardial infarction

**DOI:** 10.1515/med-2026-1558

**Published:** 2026-10-30

**Authors:** Zhaolin Yang, Heyu Meng, Jianjun Ruan, Zhiqi Yao, Fanbo Meng

**Affiliations:** Department of Cardiology, China-Japan Union Hospital of Jilin University, Jilin University, Changchun, China; Department of Endocrinology, China-Japan Union Hospital of Jilin University, Jilin University, Changchun, China

**Keywords:** NEO-1, acute myocardial infarction, coronary atherosclerosis, inflammatory marker, peripheral blood

## Abstract

**Objectives:**

This study elucidated the association between acute myocardial infarction (AMI) and Neogenin-1 (NEO-1) using proteomics to identify NEO-1 as a promising circulating biomarker for AMI risk prediction.

**Methods:**

A total of 107 participants were included in the analysis, comprising 45 control subjects without coronary heart disease (non-coronary heart disease [NCHD]; as a control group) and 62 patients with AMI. Furthermore, mRNA levels were elucidated *via* RT-qPCR, whereas the protein content was measured using enzyme-linked immunosorbent assay (ELISA). Lastly, the acquired data were elucidated and compared.

**Results:**

The data showed that relative to the NCHD group, the AMI group’s NEO-1 mRNA level in peripheral blood (PB) mononuclear cells was 1.47 times higher (p<0.05), whereas NEO-1 protein levels were 1.50 times higher (p<0.05). Furthermore, in AMI patients, the expression of NEO-1 was not related to smoking, total cholesterol, alcohol consumption, triglycerides, age, high-density lipoprotein cholesterol, hypertension, or platelets. Moreover, Logistic regression assessment revealed that AMI was associated with elevated NEO-1 expression and enhanced fasting blood glucose.

**Conclusions:**

This study revealed that relative to the NCHD patients, AMI patients had elevated PB NEO-1 expression, which was an independent risk factor for AMI. These findings indicate that NEO-1 expression is a potential bio-index for AMI.

## Introduction

The primary cause of acute myocardial infarction (AMI) is the thrombotic occlusion mediated by atherosclerotic plaque rupture in a coronary artery, which promotes prolonged ischemia in the affected myocardial region, ultimately causing cardiomyocyte necrosis [1]. This procedure relies on the location and magnitude of the infarction, requiring the complete involvement of the inflammatory response, which may instigate a cascade of complications [2]. Furthermore, various inflammatory factors and cytokines are also associated, such as TNF-α, interleukins (IL)-1, and IL-6, which essentially mediate the inflammatory cascade [3]. The implementation of percutaneous coronary intervention (PCI) has markedly enhanced treatment efficacy for individuals with acute coronary syndrome (ACS). However, patients continue to experience the risk of primary adverse cardiovascular events post-surgery. Early diagnosis of the onset of myocardial infarction (MI) and timely intervention could markedly improve patient prognosis [4]. Peripheral blood (PB) is the primary testing method for MI due to its ease of acquisition compared to tissue biopsies, facilitating access to inflammatory chemicals and cells involved in the MI inflammatory process [5]. Several PB MI markers are based on inflammatory indicators, such as for ACS patients, the potential predictive value of the platelet-to-lymphocyte ratio (PLR), and the expression levels of pro-inflammatory factor Galectin-3 (Gal-3) in PB are employed as biomarkers [6], 7].

Our previous study employed proteomic methods to determine proteins expressed in the early AMI stages [8]. It was observed that Neogenin-1 (NEO-1) levels were substantially elevated in AMI patients relative to controls, indicating an association between AMI and elevated PB NEO-1 levels. Based on the aforementioned background, this study proposes the following hypotheses: The expression level of NEO-1 in peripheral blood of AMI patients is significantly higher than that of non-coronary heart disease patients, and elevated NEO-1 expression is independently associated with the occurrence of AMI. Accordingly, the core question addressed by this study is whether the peripheral blood NEO-1 expression level can serve as a potential biomarker for predicting acute myocardial infarction (AMI). Therefore, this study analyzed a large sample size to corroborate the potential correlation between AMI patients and increased NEO-1 expression in PB. Enzyme-linked immunosorbent assay (ELISA) was carried out to validate the precision of the proteomic findings, while correlation and logistic regression assessments were conducted to investigate the association of NEO-1 with AMI, as well as the potential of employing NEO-1 level as a biomarker for AMI. The findings will provide novel insights into the expression of the NEO-1 gene in AMI and its prospective applications in treatment and diagnosis.

## Materials and methods

### Study population

The study cohort consisted of 107 hospitalized patients (admitted to the Department of Cardiology), including 62 individuals diagnosed with AMI and 45 individuals with non-coronary heart disease (NCHD). All the participants received a 12-lead electrocardiogram and related auxiliary tests at admission. The diagnosis of AMI was made according to the universal definition of myocardial infarction, as outlined in the 2020 clinical practice guidelines released by the European Society of Cardiology [9]. Coronary angiography revealed critical stenoses and complete occlusions of the left main coronary artery, the right main coronary artery, and their major branches, especially the left anterior descending and circumflex arteries, thus confirming the diagnosis of AMI. Exclusion criteria for the AMI group comprised patients (1) lacking AMI-associated serum markers such as troponin and myoglobin; (2) experiencing MI arising from conditions that lead to an insufficient balance between myocardial oxygen supply and demand; (3) presenting with stent thrombosis or prior percutaneous coronary intervention; and (4) having undergone coronary artery bypass grafting.

### Experimental process

Eligible AMI patients and NCHD patients were enrolled, and their PB was collected for further analysis. NEO-1 protein and mRNA expressions in AMI groups were evaluated via ELISA and RT-PCR, respectively. Moreover, logistic regression analysis was also carried out using SPSS 27.0 software (Figure 1).

**Figure 1:**
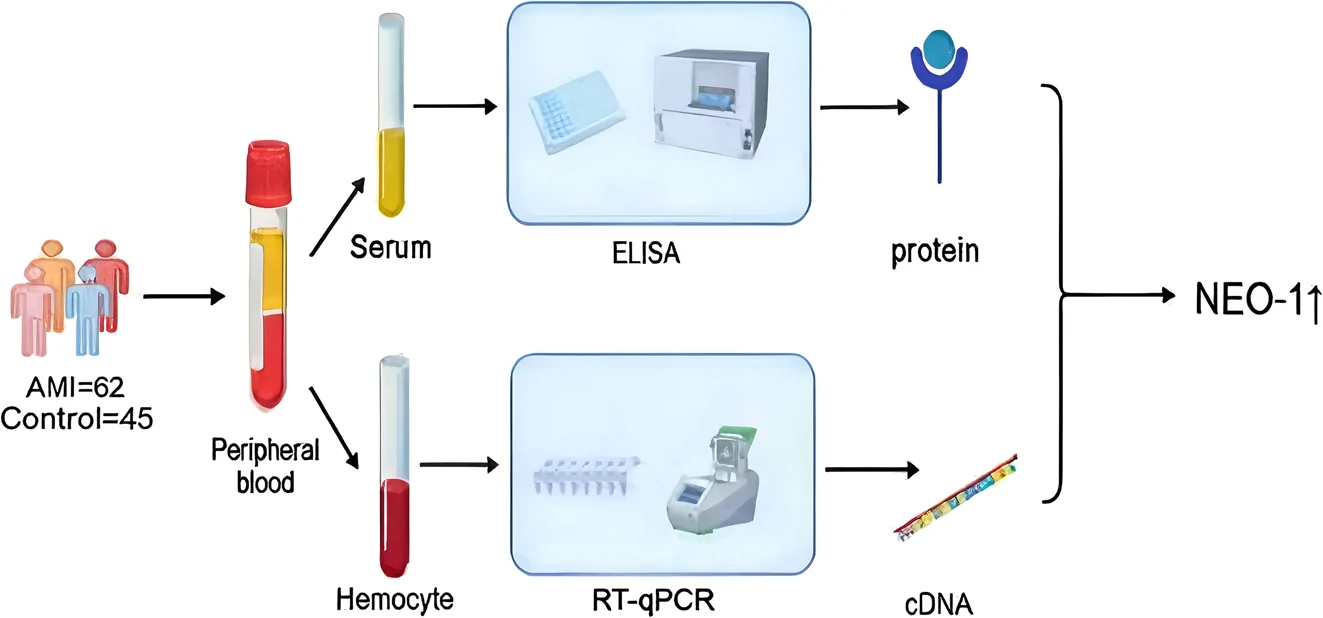
Diagrammatic representation of the experimental scheme.

Peripheral blood samples from 62 AMI and 45 NCHD patients were collected and centrifuged. Serum was collected from the upper layer to assess protein content via ELISA, while the lower cell layer was employed to detect mRNA levels via RT-qPCR.

### RT-qPCR

#### Peripheral blood mononuclear cells (PBMCs) isolation and processing

Peripheral blood samples (6 mL) were drawn from participants after an 8 h fasting period into vacuum tubes containing EDTA anticoagulant, with subsequent preservation at 4 °C for further processing. Within 4 h, PBMCs were isolated with Lymphoprep lymphocyte separation medium (Stem Cell Technologies, Vancouver, Canada). Equal volumes of Lymphoprep, saline, and fresh anticoagulated blood were mixed, followed by their centrifugation for 20 min at 1,000 g to acquire four layers of serum, milky mononuclear cells, clear liquid, and red blood cells (from top to bottom). The mononuclear cell layer was aspirated for further assays.

#### Extraction of RNA and synthesis of cDNA

The RNA contents of PBMCs were extracted by means of the Total RNA Extraction Kit (Tiangen, Beijing, China), according to the manufacturer’s protocol, with subsequent concentration determination by spectrophotometric analysis. RNA quality was verified according to two spectrophotometric criteria: A260/A280 ratios between 1.7–2.1 and A260/A230 ratios exceeding 2.0. Qualified RNA samples were reverse transcribed into complementary DNA using the manufacturer’s protocol (Tiangen, Beijing, China), with subsequent storage of cDNA at −80 °C until further analyese.

#### qPCR analysis

Before amplification, cDNA was diluted 20-fold. For amplification, qPCR synthesis premix and SYBR Fluorescence Quantification Kit (Sangon, Shanghai, China) were employed under the following reaction conditions: initial denaturation at 95 °C for 3 min, followed by 40 cycles of 95 °C for 3 s, 60 °C for 30 s, and 72 °C for 20 s. The ABI 7500 Fast Real-Time PCR System (Applied Biosystems, USA) was utilized for acquiring melting and dissociation curves for temperatures ranging between 60 and 95 °C. GAPDH was employed as an endogenous control, and the target gene was NEO-1. The 2-ΔΔCT method was applied to identify relative gene expression. Table 1 indicates primer sequences.

**Table 1:** The sequences of the designed primers employed for the analysis of qPCR.

Genes	Genes primer sequence (5′-3′)
GAPDH	Fa: TGGGTTATTGAGCCTGTTG Rb: GGAGTCCGCTTTAGGTGTTC
NEO-1	Fa: GTCTCCTCTGACTTCAACAGCG Rb: ACCACCCTGTTGCTGTAGCCAA

Fa, upstream primer; Rb, downstream primers.

### ELISA

The PB samples were collected and processed as mentioned in Section 2.3.1 (Peripheral Blood Mononuclear Cells Isolation and Processing). The top serum layer was aspirated and kept at −80 °C for ELISA. Briefly, the NEO-1 protein levels were determined using the method described in a commercial ELISA kit (Invitrogen Biotechnology Co., Shanghai, China). Absorbance (OD) measurements were performed at a wavelength of 450 nm with a microplate reader. The concentration of NEO-1 was interpolated from a standard curve constructed by linear regression analysis of the reference standards. The resulting calibration curve (R^2^=0.9946) enabled precise quantification across the dynamic range of 0.625–20 ng/mL.

### Statistical assessments

All the data were elucidated via SPSS v27.0. The median and interquartile range were employed to represent non-normally distributed data (p≤0.1), whereas their intergroup differences were evaluated with the independent samples rank-sum test. The intergroup differences of normally distributed data (p>0.1) were assessed via an independent samples t-test, and the values were depicted as mean ± SD. For categorical data presentation, frequencies were employed, and the chi-square (χ^2^) test was carried out to assess their intergroup differences. Binary logistic regression analysis was conducted to further evaluate the association between established risk factors and AMI incidence. Statistical significance was defined as a two-tailed p-value less than 0.05.

### Ethical approval and informed consent

All protocols conformed to the Declaration of Helsinki (as revised in 2013). The study strictly adhered to local regulations and institutional requirements and was approved by the Ethics Committee of China-Japan Union Hospital of Jilin University (approval No. 2016WJW017; approval date: November 24, 2016). Informed consent was obtained from all individuals included in this study, or their legal guardians or wards.

## Results

### Baseline data assessment

Patient’s demographic and clinical data, including total cholesterol, smoking, alcohol consumption, age, fasting blood glucose (FBG), diabetes, high-density lipoprotein cholesterol (HDL-C), hypertension, low-density lipoprotein cholesterol (LDL-C), triglycerides, and platelets, were compared. The data indicated marked differences in diabetes, FBG, and LDL-C (p<0.05) between the two groups (Table 2).

**Table 2:** Baseline data of the participants.

Clinical indicators	Control (n=45)	AMI (n=62)	t/z/x^2^	p-Value
Age	62.00 (52.00–68.50)^a^	64.00 (55.75–69.00)^a^	−0.88	0.38
TG, mmol/L	1.38 (0.95–1.94)^a^	1.61 (1.13–2.19)^a^	−1.19	0.24
TC, mmol/L	4.42 ± 1.19	5.11 (3.86–5.80)^a^	−1.65	0.10
HDL, mmol/L	1.15 ± 0.30	1.15 ± 0.29	0.08	0.94
LDL, mmol/L	2.73 ± 0.96	3.15 ± 0.96	2.25	0.03
Fasting blood-glucose, mmol/L	5.51 (4.99–6.43)^a^	6.74 (5.37–8.97)^a^	−3.34	<0.01
Platelet, ×10^9^/L	228.00 (184.50–257.00)^a^	240.00 (200.50–289.00)^a^	−1.05	0.30
Hypertension, %	35.50	50.00	2.21	0.14
Diabetes, %	8.80	27.40	5.68	0.02
History of smoking, %	31.10	50.00	3.82	0.05

^a^Data presented as Median (IQR) for non-normal distribution.

### NEO-1 protein level

The linear regression equation for the standard was y=0.021x + 0.0671 (R^2^=0.9946), which was employed to assess protein concentration. Peripheral blood serum ELISA results showed that the NEO-1 protein concentration in AMI patients was 71.33 (61.18–82.97) ng/mL, compared to 47.64 (37.61–56.09) ng/mL in the control group. Consequently, the AMI patient group demonstrated a 1.5-fold elevation (p<0.05) in circulating NEO-1 protein levels compared to the NCHD control cohort (Figure 2).

**Figure 2:**
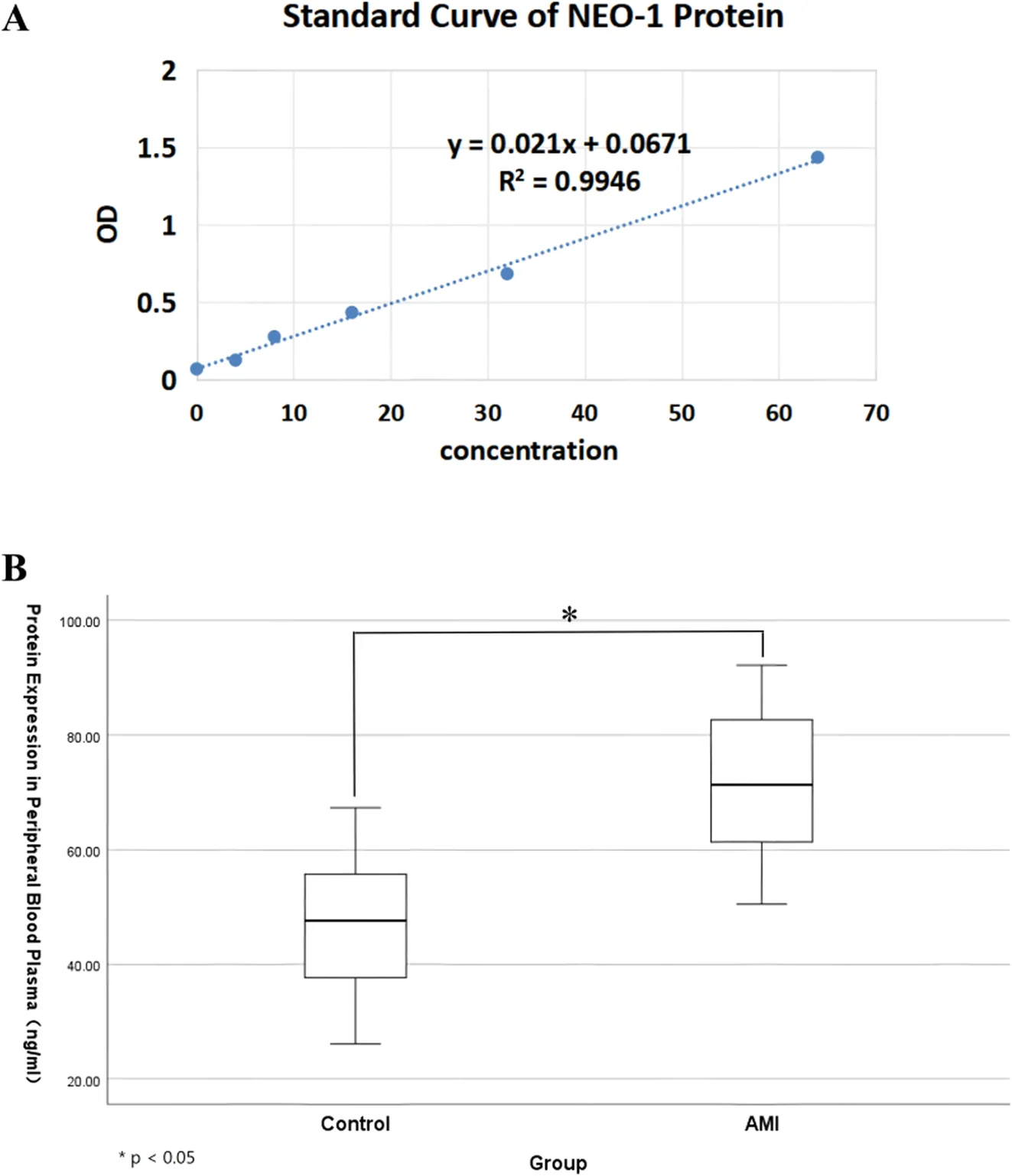
ELISA results: (A) standard curve of NEO-1 protein. (B) NEO-1 protein expression in peripheral plasma, *p<0.05.

### NEO-1 mRNA level

RT-qPCR analysis was performed using peripheral blood mononuclear cells (PBMCs). Relative gene expression was calculated using the 2ˆ-ΔΔCTˆ method. RT-qPCR revealed that NEO-1 mRNA content was substantially elevated in the AMI group (1.47 times, p<0.05). The 2-ΔΔCT value for the AMI and NCHD groups was 1.58 (0.54–3.17) and 1.08 (0.69–1.57), respectively (Figure 3).

**Figure 3:**
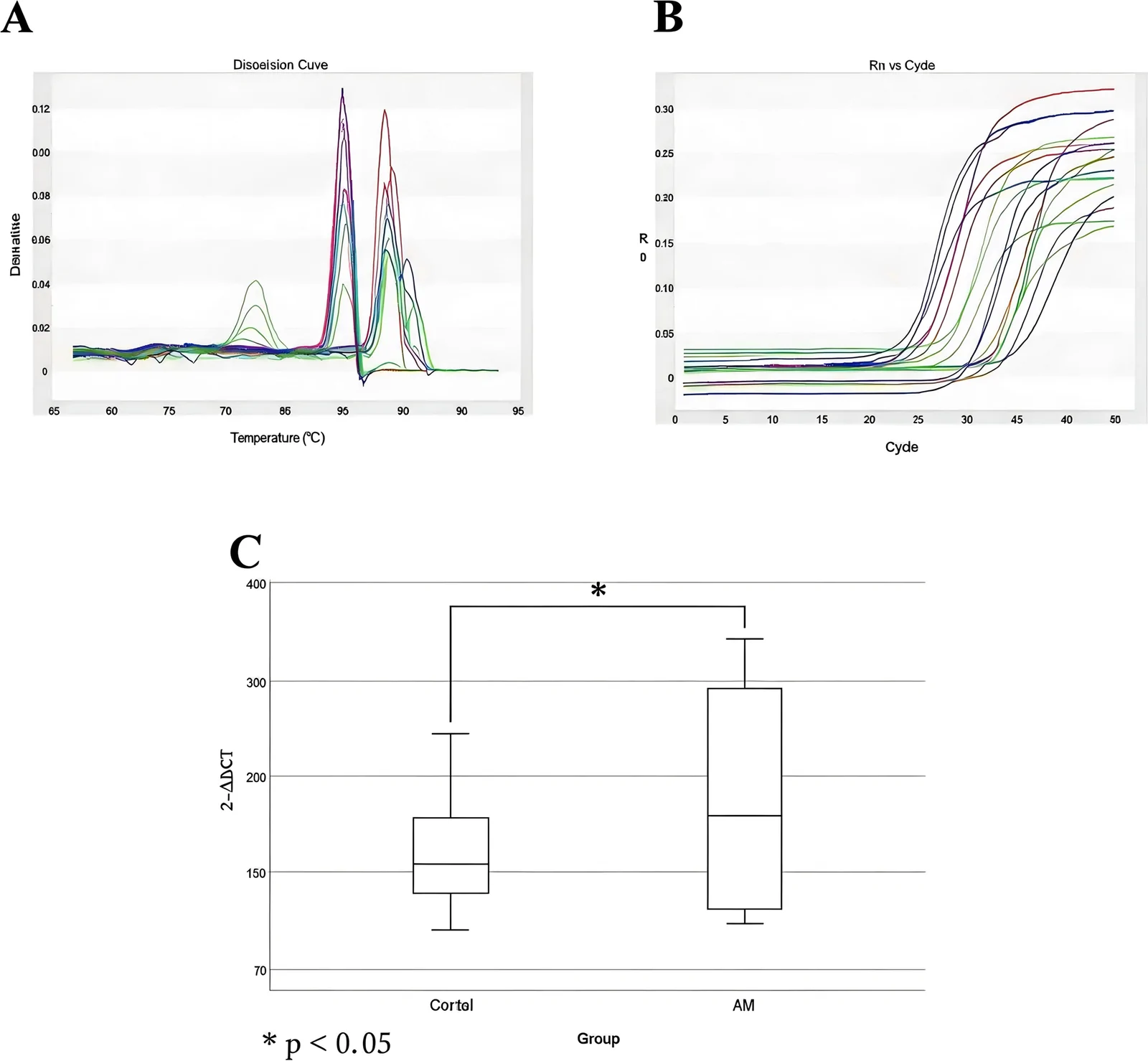
The analysis of qRT-PCR: (A) the amplification plot associated with the NEO-1 gene; (B) melt curve associated with the NEO-1 gene; (C) comparative mRNA expression in PBMCs, *p<0.05.

### Correlation between sample characteristics and NEO-1 protein content

Given the statistically significant variations (p<0.05) in NEO-1 protein concentrations between AMI and NCHD groups across multiple demographic and clinical parameters (Table 3), particularly diabetes status, FBG, and LDL-C, participants were stratified into subgroups based on FBG levels (normal <7 mmol/L; diabetic ≥7 mmol/L), diabetic status, and LDL-C concentrations (normal: <4.1 mmol/L; hypercholesterolemia ≥4.1 mmol/L). The correlation analysis revealed a significant association (p<0.05) between the concentrations of LDL-C and FBG and NEO-1 protein levels.

**Table 3:** The correlation analysis.

Groups	NEO-1 expression	Z	p-Value
High LDL-C group	69.12 ± 16.09	−2.18	0.03
Normal LDL-C group	60.05 ± 16.77		
High fasting blood glucose group	66.29 ± 13.74	−2.10	0.04
Normal fasting blood glucose group	59.26 ± 17.87		
Diabetes group	62.89 (58.51–76.49)^a^	−1.10	0.27
Diabetes-free group	60.57 ± 17.53		

^a^Data presented as Median (IQR).

### Logistic regression analysis of AMI-associated factors

An optimal NEO-1 cut-off concentration of 55.43 ng/mL was established for discriminating AMI patients based on the maximum Youden index, defined as (sensitivity + specificity − 1). The participants were subdivided based on NEO-1 levels into high (NEO-1 content >55.43) and low (content ≤55.43) groups. Logistic regression test revealed that high NEO-1 protein and FBG levels were linked with AMI. This indicates that elevated NEO-1 protein levels can act as an independent risk factor for AMI onset, elevating the AMI risk by 1.16 times (p<0.05). Elevated FBG emerged as an independent predictor of AMI in our cohort, exhibiting a 4.71-fold increase in AMI risk (p<0.05) after adjusting for confounding variables (Table 4).

**Table 4:** Results of logistic regression analysis identifying independent AMI risk factors.

	B	Standard variation	Wald	Degree of freedom	p-Value	OR	95 % CI
Protein expression of NEO-1	0.14	0.03	21.51	1.00	<0.01	1.16	1.09–1.23
High fasting blood glucose group	1.55	0.65	5.61	1.00	0.02	4.71	1.31–16.96
History of smoking	1.10	0.62	3.17	1.00	0.08	3.00	0.90–10.03
Hypertension	0.07	0.60	0.01	1.00	0.91	1.07	0.33–3.45
High LDL-C	0.12	0.94	0.02	1.00	0.90	1.13	0.18–7.07

AMI, acute myocardial infarction; OR, odds ratio; CI, confidence interval.

## Discussion

This study aims to address the core question: whether the peripheral blood NEO-1 expression level can serve as a potential biomarker for predicting acute myocardial infarction (AMI). The results indicated that NEO-1 mRNA and protein levels were notably enhanced in AMI individuals relative to the NCHD patients. Furthermore, the AMI patients’ mRNA level was 1.47 times that of the NCHD group, while the protein level was 1.50 times higher. Moreover, logistic regression assessment revealed that AMI was related to increased NEO-1 and FBG levels. In addition, enhanced NEO-1 expression was identified as an independent AMI risk factor, suggesting the potential to serve as a predictor of AMI.

Previous proteomic data indicated that NEO-1 protein levels are markedly higher in AMI patients than in normal individuals. While initial investigations indicated a correlation between NEO-1 and atherosclerosis, only a few studies have indicated the association between NEO-1 levels and AMI. Therefore, this research study evaluated the possible correlation between NEO-1 levels and AMI using a large sample size to corroborate the previous proteomic findings. Furthermore, RT-qPCR and ELISA were conducted to measure NEO-1 mRNA and protein concentrations in the PB of AMI and NCHD patients, respectively. The expression level of NEO-1 was significantly (p<0.05) increased in AMI patients when compared with the NCHD group, with relative increases of 1.47-fold and 1.50-fold, respectively.

NEO-1 is essentially involved in various inflammatory processes, such as hepatic ischemia-reperfusion injury, acute peritonitis, and lung injury. Furthermore, it also improves blood-brain barrier dysfunction after subarachnoid hemorrhage [10], [11], [12], [13]. It was initially identified by Vielmetter, Kayyem, Roman, and Dreyer during their investigation of new proteins implicated in brain development [14]. Fluorescence *in situ* hybridization revealed that the chromosomal location of NEO-1 was 15q22.3-q23, situating adjacent to a locus linked with Bardet-Biedl syndrome [15]. The NEO-1 gene was first identified based on its sequence homology to the deleted in colorectal cancer (DCC) gene. Its transmembrane receptor protein interacts with repulsive guidance molecule (RGM) ligands and netrins, guiding axonal navigation during neural development [16]. The interaction between axon guidance molecules and their receptors mediates carcinogenesis via cell survival and apoptosis, angiogenesis, terminal cell differentiation, and cell localization and migration [17], 18]. As a member of the immunoglobulin superfamily, the NEO-1 receptor exhibits a characteristic single-pass transmembrane topology. Its structure is defined by an extracellular region containing four Ig-like and six fibronectin type III-like domains, a solitary transmembrane helix, and an intracellular domain [19]. The intracellular domain contains 3 conserved motifs responsible for signaling [20]. Functionally, NEO-1 is similar to Notch or APP receptors. After ligand binding, γ-secretase cleaves NEO-1’s intracellular domain to release the neogenin intracellular domain (NeICD), which is imported/exported to the nucleus and regulates gene transcription. NeICD also binds histone acetyltransferases; however, the variables that activate transcription within the NeICD/DNA complex have yet to be identified [21]. As a key modulator of neuroinflammation, the repulsive guidance molecule (RGM) family comprises glycosylphosphatidylinositol (GPI)-anchored glycoproteins containing an N-terminal signal peptide. Research has documented elevated expression of the RGMa isoform in response to ischemic stroke in patients and experimental spinal cord injury in rats. The ensuing signaling through the RGM-neogenin axis is now recognized as a major contributor to the pathology of neuroinflammatory diseases, with its activity levels directly correlating with the progression of neurological deficits [22], [23], [24]. Netrins are axon chemotactic factors with chemotactic attractive or repulsive guidance activities. DCC and neogenin-like proteins serve as receptors for netrins. Mouse stroke model has indicated elevated gene expression of DCC, a netrin chemotactic receptor in neurons and astrocytes in the nervous system, as well as elevated NEO gene expression in endothelial cells [25]. Originally characterized for their roles in neuronal guidance, Netrin isoforms 1–4 have been increasingly implicated in vascular development and pathophysiology [26], expanding their functional significance beyond the nervous system. However, the only Netrin protein family member described in the cardiovascular system to date is Netrin-1 [27]. Netrin-1 interacts with receptors DCC/NEO/UNCoordinated-5 (UNC5) family members, exerting diverse effects. Moreover, it modulates inflammation and leukocyte infiltration, indicating its association with immune responses [28].

A prevalent heart condition, coronary heart disease, is mainly caused by narrowing, hardening, or occlusion of the coronary arteries, which results in the development of MI. These pathophysiological phenomena, driven by atherosclerotic plaque formation involving lipid accumulation and inflammatory cell infiltration within the arterial wall, ultimately compromise blood flow to the myocardium and induce ischemic injury [29]. Coronary atherosclerosis is a prevalent pathophysiological mechanism in ACS. Atherosclerosis is a chronic inflammation impacting medium and large arteries [30]. Chronic inflammation is caused by inflammation and lipids [31], which eventually leads to the formation of various plaques. The type of plaque determines the occurrence of different cardiovascular events. Pathological studies of MI have indicated a correlation between plaque rupture and adverse plaque characteristics, such as large necrotic core, positive remodeling, microcalcification, and thin fibrous cap. These plaque characteristics are markedly associated with AMI [32]. Recent research has revealed that AMI may involve a series of inflammatory processes. Inflammatory chemicals implicated in these processes induce aberrant platelet activation and coagulation, as well as promote endothelial dysfunction. In these processes, inflammatory cells, including macrophages and neutrophils, along with their produced cytokines, significantly contribute to the aggravation of inflammation [33]. A marked number of studies indicate that platelet aggregation, rupture of unstable atherosclerotic plaques, and thrombosis can cause occlusion or vascular stenosis, which may result in the development of acute cardiovascular diseases [34]. In the last few decades, several studies have indicated the significance of inflammation in the atherosclerosis onset, progression, and destabilization [35]. The current research identified elevated NEO-1 expression as a potential independent risk factor for AMI, potentially mediated through its role in promoting pro-inflammatory pathways.

The pathogenesis of atherosclerosis, a chronic inflammatory disorder of the arterial wall, begins with the deposition of oxidized low-density lipoprotein (ox-LDL) in the subendothelial space, which subsequently induces endothelial activation and initiates a sustained inflammatory response [36]. Activated endothelial cells release cytokines, chemokines, and other inflammatory mediators, which continuously recruit monocytes and other inflammatory cells to the vascular wall [37]. Multiple harmful stimuli (smoking, abnormal hemorheology, hypertension, hyperlipidemia, viruses, etc.) can cause endothelial injury. It has been observed that endothelium maintains a delicate balance between vasoconstriction and vasodilation, as well as anticoagulant and procoagulant activities [38]. Furthermore, injurious stimuli induce the endothelium to upregulate nuclear factor kappa-light-chain-enhancer of activated B cells (NF-κB) transcription factor and release various substances that elevate leukocyte adhesion to the endothelium, such as vascular cell adhesion molecule (VCAM-1 and ICAM-1), E-selectin, endothelin, angiotensin II, and procoagulant factors [39]. NF-κB is a primary signaling system that modulates inflammation, cell survival, immunity, and proliferation, significantly contributing to vascular disorders such as atherosclerosis, MI, and heart failure [40].

Recent studies demonstrate that NEO-1 serves several functions within the cardiovascular system, specifically related to atherosclerosis and post-MI cardiac remodeling. NEO-1 expression in various cell types demonstrates distinct biological functions. NEO-1 expression in endothelial cells promotes key pro-atherogenic processes, thereby accelerating atherosclerotic lesion development. Upregulated NEO-1 expression potentiates ox-LDL-induced inflammatory signaling by enhancing NF-κB activation. This phenomenon results in the transcriptional upregulation of NF-κB-dependent adhesion molecules and cytokines, which subsequently amplify monocyte adhesion and transmigration across the endothelium. This cascade intensifies the inflammatory milieu within the arterial wall and accelerates atherogenesis. NEO-1 blocking antibodies can attenuate atherosclerotic lesions in animal models [41], [42], [43]. NEO-1 expression in macrophages participates in post-MI cardiac remodeling. IFN-γ can activate the JAK1/2-STAT1 pathway to induce macrophage activation, leading to the transformation of macrophages into a pro-inflammatory phenotype [44]. NEO-1 in macrophages modulates macrophage differentiation into a pro-inflammatory phenotype by inhibiting the JAK1-STAT1 signaling cascade, thereby mitigating the inflammatory response, reducing cardiomyocyte death, and enhancing cardiac function. Lysophosphatidylcholine (LPC) is a major lipid in ox-LDL and serves as a potent inflammatory mediator. It induces macrophage chemotaxis, upregulates the generation of proinflammatory cytokines, and drives chronic inflammation, such as atherosclerosis. The increase in LPC caused by anti-NEO-1 also indicates that NEO-1 deficiency is detrimental to apoptotic cell clearance and inflammation resolution after MI. Moreover, NEO-1 deletion enhances inflammation and ventricular remodeling after MI [45], 46].

## Limitations

This retrospective study indicated that a high NEO-1 level was related to AMI, suggesting its potential as an AMI diagnostic marker. The findings provided novel strategies and targets for AMI prevention and treatment by highlighting potential intervention targets. The interpretation of these results; however, must be tempered by an awareness of the study’s limitations. This investigation had a relatively small sample size, which might have some errors. Furthermore, a prospective design should be employed in the future. Moreover, the underlying mechanism of elevated NEO-1 in AMI remains unclear and requires further investigation.

## Conclusions

In summary, this study revealed that relative to NCHD patients, AMI patients had increased NEO-1 mRNA and protein levels. Furthermore, its increased levels can significantly lead to elevated AMI risk by promoting inflammation. Thus, it was inferred that high expression of NEO-1 makes it as an independent risk factor associated with AMI, and, therefore, it can be effectively employed as a biomarker for AMI diagnosis.
